# The effect of eggshell thickness on hatchability of quail eggs

**DOI:** 10.14202/vetworld.2017.1114-1117

**Published:** 2017-09-23

**Authors:** Oguz Fatih Ergun, Umut Sami Yamak

**Affiliations:** Department of Animal Science, Agricultural Faculty, Ondokuz Mayis University, Samsun, Turkey

**Keywords:** eggshell thickness, hatchability, incubation, quail, ultrasound

## Abstract

**Background and Aim:**

The aim of the successful incubation period is to achieve maximum health chicks in each batch. Therefore, all factors affecting incubation have to be investigated in detail. This study investigated the effect of eggshell thickness on hatchability of quail eggs.

**Materials and Methods:**

A total of 1415 eggs were collected from the same flock at the ages of 23 and 41 weeks. Two different incubations were performed at these eggs. Eggshell thicknesses of all eggs were determined with an ultrasonic gauge before incubation. Incubation period was applied as for 18 days. After 15 days of incubation, eggs were transferred to hatching machine. Eggs were classified as thin-, medium-, and thick-shelled according to eggshell thickness values.

**Results:**

Eggshell thicknesses were ranged between 0.24 and 0.36 mm, and the differences between the hatching rates of thickness values were not found significant. Hatchability of thin-, medium-, and thick-shelled eggs was found as 69.2%, 69.4%, and 82.4% for Experiment 1. These values were as 87.8%, 89.2%, and 91.9% for Experiment 2, respectively. Similar to eggshell thickness frequencies, the differences between hatching rates of eggshell thickness groups were found insignificant.

**Conclusion:**

Results of this study showed that eggshell thickness does not affect hatchability.

## Introduction

The role of eggshell thickness on hatchability has been comprehensively investigated in the previous studies. The studies which assessed the thickness indirectly, mostly found a relation between eggshell thickness and hatchability. In these studies, researchers have assessed eggshell thickness according to egg-specific gravity [1], with a logarithm that used egg weight [2] or measured shell thickness after hatch [3]. Bennet [1] found that eggs with thin shells had hatchability rates between 3% and 9% lower than eggs with thick shells, whereas Tsarenko [4] reported a 30% difference in hatchability rates between thin- and thick-shelled eggs.

A number of studies examining hatchability in different poultry species including turkey and geese found hatchability of thick-shelled eggs to be 20-40% higher than that of thin-shelled eggs [5], although a study by Andrews [6] found the hatchability of turkey eggs to be higher for eggs with thinner shells. Despite their differences in the findings, all of these studies reported eggshell thickness to have an effect on egg hatchability.

On the other hand, the studies which measured thickness using an ultrasound gauge found any relation between eggshell thickness and hatchability in different poultry species. No significant differences were observed between hatching rates and eggshell thickness of chicken eggs [7]. Similar results were found in partridge [8], guinea fowl, and pheasant eggs [9]. Furthermore, the effect of eggshell thickness on hatching time was found insignificant [8]. These studies showed that once the chick embryo has completed its development, even thick- or thin-shelled eggs may hatch successfully. Therefore, same ultrasound method was used to observe the effect of eggshell thickness on hatchability of quail eggs in this study.

## Materials and Methods

### Ethical approval

This study was conducted at the experimental farm of the Ondokuz Mayis University, Agricultural Faculty. The care and use of animals were in accordance with laws and regulations of Turkey and approved by the Ethical Committee of Ondokuz Mayis University (License number: 2015-31).

### Material

Two different incubations were conducted with the eggs, collected from the breeder quail (Coturnix Coturnix Japonica) flock of farm. In the first incubation, 1000 eggs were collected in 7 days from the flock at 23 weeks of age, and 415 eggs were collected in 3 days from the same flock at 41 weeks of age. In both incubations, all eggs were numbered and weighed, and shell thickness was measured on the blunted edge with an eggshell thickness gauge (ORKA Tech. Ltd., Israel) that uses precision ultrasound to measure thickness without breaking the egg and is accurate to within 0.01 mm. The total incubation period was 18 days. Eggs were incubated for 15 days in an incubator set at 37.5°C and 60% relative humidity and then transferred to a hatching machine set at 36.5°C and 70% relative humidity. After the 18-day incubation period, all unhatched eggs were broken open and infertile eggs, and embryonic deaths were identified. Infertile eggs (n=309 for Experiment 1 and n=95 for Experiment 2) were not used in the calculation of hatching rates. Hatching rates were calculated as the percentage of hatched chicks to fertilized eggs.

**Figure-1:**
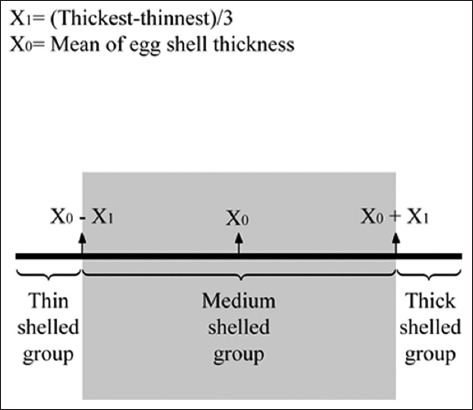
Classification method of eggshell thickness groups.

The thinnest and thickest eggshell thickness values of the eggs were determined for each experiment. The difference between thickest and thinnest eggs was divided into three (X_max_−X_min_/3). This value was added to mean eggshell thickness to determine the range of thick shell group and deducted from mean eggshell thickness to determine the range of thin-shell group [7]. The eggs were classified to three eggshell thickness groups (thin, medium, and thick) with this method (Figure-1). Hatching rates were given for eggshell thickness groups and for each thickness value in both two experiments.

### Statistical analysis

All statistical analysis was performed using SPSS Software Version 20.0 licensed to Ondokuz Mayis University [10]. Frequency analysis was performed for eggshell thickness groups using Tukey’s Hinges test [11], and eggshell thickness groups were formed according to frequency percent of groups. Kruskal–Wallis tests (Siegel and Castellan, 1988) were used to examine the effects of eggshell thickness (by percentile group as well as by thickness value) on hatchability. Kendall’s tau correlation analysis (Siegel and Castellan, 1988) was used to assess relationships between eggshell thickness and hatchability because of discrete data. The effect of eggshell thickness value on hatchability was analyzed using the Chi-square test [12]. A difference of p<0.05 was considered statistically significant.

## Results

Eggshell thickness of fertilized quail eggs (n=691) ranged between 0.24 and 0.35 mm in Experiment 1. In Experiment 2, eggshell thickness values of fertilized eggs (n=320) ranged between 0.24 and 0.36 mm. Hatching rates for both two experiments were given in Table-1. Eggshell thickness value had an insignificant effect on hatching rates (p=0.236 for Experiment 1 and p=0.965 for Experiment 2).

**Table-1:** Hatching rates of eggshell thickness values in two different experiments.

	Experiment 1			Experiment 2		
	
	Eggshell thickness value (mm)	n	Hatching rate (%)	Eggshell thickness value (mm)	n	Hatching rate (%)
	0.24	3	100	0.24	7	71.4
	0.25	33	72.7	0.25	18	83.3
	0.26	113	73.5	0.26	23	91.3
	0.27	182	65.4	0.27	42	90.5
	0.28	173	69.9	0.28	61	86.9
	0.29	98	67.3	0.29	64	87.5
	0.30	41	75.6	0.30	56	91.1
	0.31	31	64.5	0.31	51	92.2
	0.32	5	80.0	0.32	14	92.9
	0.33	3	66.7	0.33	11	90.9
	0.34	6	100	0.34	10	90.0
	0.35	3	69.6	0.35	1	100
				0.36	1	100
Mean	0.28		75.43	0.29		89.85
p	0.236			0.965		

Eggs were classified into groups according to shell thickness. Thickness groups were consisted as thin (≤0.28 mm), medium (0.29-0.31 mm), and thick (≥0.32 mm) for Experiments 1 and 2. Out of a total of 691 eggs, 331 eggs were classified as thin-shelled, 343 as medium-shelled, and 17 as thick-shelled for Experiment 1. These numbers were as 79 (thin), 207 (medium), and 34 (thick) for Experiment 2. Hatching rates for thin-, medium-, and thick-shelled quail eggs of Experiment 1 were 69.2%, 69.4%, and 82.4%, respectively (Table-2). The differences in hatching rates between groups were not significant (p=0.543). Hatching rates for thin-, medium-, and thick-shelled quail eggs of Experiment 2 were 87.8%, 89.2%, and 91.9%, respectively (Table-2). Similar to the results of Experiment 1, the differences in hatching rates between groups were not significant for Experiment 2 (p=0.462).

**Table-2:** Hatching rates of eggshell thickness groups in two different experiments.

Eggshell thickness group	Experiment 1		Experiment 2	
	
	n	Hatching rate (%)	n	Hatching rate (%)
Thin-shelled eggs	331	69.2	79	87.8
Medium-shelled eggs	343	69.4	207	89.2
Thick-shelled eggs	17	82.4	34	91.9
p	0.543		0.462	

p: Type I error rate. The differences are non-significant while P>0.05

## Discussion

Mean eggshell thickness has been reported to range between 0.17 and 0.30 mm for quail eggs [13,14]. Our study found the mean eggshell thickness of quail eggs at 23 and 41 weeks of age to be 0.28 and 0.29 mm, respectively. Hatchability rates were assessed as the ratio of hatched chicks to fertilized eggs. Overall, hatching rates were 75.43% for Experiment 1 and 89.85% for Experiment 2 (Table-1). Eggs were stored for 7 days in Experiment 1 and 3 days in Experiment 2. Longer duration of storage could affect the hatching rate of eggs in Experiment 1. Seker *et al*. [15] reported the mean hatchability of quail eggs 90.39% in 3 days storage and 67.96% in 7 days storage. Although the difference between two experiments, hatching rates obtained in the current study were similar to reported hatchability rates of quails between 58.33% and 93.33% [15,16]. Hatching rates were separately calculated for each thickness value of quail eggs in this study. Hatching rates ranged between 64.5% and 100% in Experiment 1 and 71.4 and 100% in Experiment 2. The differences between hatching rates by eggshell thickness values were not statistically significant for Experiment 1 (p=0.236) and Experiment 2 (p=0.965). Similar to these findings, no significant differences were found in hatching rates of guinea fowl and pheasant eggs by eggshell thickness [9].

To determine the effect of eggshell thickness on hatchability, this study grouped eggs into thin-, medium-, and thick-shelled groups based on calculations made using fertilized eggs only. Whereas hatching rates of thick-shelled eggs were higher than hatching rates of thin- and medium-shelled eggs in both two experiments. In both cases, the differences in hatching rates between eggshell thickness groups were statistically insignificant (p>0.05).

However, most of the previous studies have shown significant differences between the hatching rates of thin- and thick-shelled eggs - although some studies report thin-shelled eggs to have higher hatching rates and others report thick-shelled eggs to have higher hatching rates in different poultry species [1,4-6,17]. The methodology used to determine eggshell thickness could be the reason for the differences in reported findings regarding the relationship between eggshell thickness and hatchability.

In contrast to the direct measurement by ultrasound used in this study, calculations based on specific gravity or egg weight all rely on indirect methods of measurement. In a study comparing various indirect methods for measuring eggshell thickness, Yamak *et al*. [18] showed that the same chicken egg could be identified as thin-shelled by one indirect method and as thick-shelled by another indirect method. These findings highlighted the importance of direct measurement. The previous studies conducted using the same direct ultrasound measurement method used in this study but with different poultry species, namely, chickens [7], partridges [8], guinea fowl, and pheasants [9] also found hatching rates to be unaffected by eggshell thickness. Moreover, the hatching time of partridge eggs was not affected by eggshell thickness [8].

## Conclusion

This study measured eggshell thickness directly using an ultrasound gauge and found no significant differences in hatching rates by eggshell thickness in quail eggs. The findings of this study were in parallel with the previous studies which assessed the shell thickness by ultrasound and investigated the relation between eggshell thickness and hatchability in different poultry species. Results showed that developed embryos could hatch successfully regardless of thickness. Further studies need to be conducted to execute the relation between eggshell thickness and developing embryo during incubation.

## Authors’ Contributions

USY planed and designed for the study. The eggs were collected and weighed, and the thicknesses were determined by OFE. Incubations were performed by OFE and USY. USY drafted and revised the manuscript. All authors read and approved the final manuscript
